# Captivity reduces diversity and shifts composition of the Brown Kiwi microbiome

**DOI:** 10.1186/s42523-021-00109-0

**Published:** 2021-07-08

**Authors:** Priscilla A. San Juan, Isabel Castro, Manpreet K. Dhami

**Affiliations:** 1 Department of Biology, Stanford University, 371 Serra Mall, Stanford, California 94305 USA; 2 Center for Conservation Biology, Stanford University, Stanford, California USA; 3 Wildlife and Ecology Group, School of Agriculture and Environment, Massey University, Palmerston North, New Zealand; 4 Manaaki Whenua – Landcare Research, 54 Gerald Street, 7608 Lincoln, New Zealand

## Abstract

**Background:**

Captive rearing is often critical for animals that are vulnerable to extinction in the wild. However, few studies have investigated the extent to which captivity impacts hosts and their gut microbiota, despite mounting evidence indicating that host health is affected by gut microbes. We assessed the influence of captivity on the gut microbiome of the Brown Kiwi (*Apteryx mantelli*), a flightless bird endemic to New Zealand. We collected wild (*n* = 68) and captive (*n* = 38) kiwi feces at seven sites on the north island of New Zealand.

**Results:**

Using bacterial 16 S rRNA and fungal ITS gene profiling, we found that captivity was a significant predictor of the kiwi gut bacterial and fungal communities. Captive samples had lower microbial diversity and different composition when compared to wild samples. History of coccidiosis, a gut parasite primarily affecting captive kiwi, showed a marginally significant effect.

**Conclusions:**

Our findings demonstrate captivity’s potential to shape the Brown Kiwi gut microbiome, that warrant further investigation to elucidate the effects of these differences on health.

**Supplementary Information:**

The online version contains supplementary material available at 10.1186/s42523-021-00109-0.

## Background

Diet, behavior, habitat type, and environmental species pools can all influence the composition and diversity of gut microbiomes [1–4]. However, few studies have investigated the impact of captivity, a severe lifestyle shift, on avian gut microbiota. Reports on nine species of parrots, red-crowned crane (*Grus japonensis*), and vultures (*Gyps fulvus* and *Neophron percnopterus*) demonstrate that captivity can impact gut microbiomes [5–7]. Fewer studies have compared wild and captive gut bacteria and fungi across spatially distinct sites that vary by climate and vegetation [8], which are expected to differ in microbial species pools, a potential source for gut microbes.

Captive rearing is often necessary to conserve populations of threatened wildlife. In the case of the Brown Kiwi (*Apteryx mantelli*), predation from introduced mammals has made it imperative for some chicks to be raised in captivity until individuals are large enough to defend themselves [9]. Although successful in increasing population size, consequences to kiwi health via modification of the gut microbiome remain largely unknown. Factors pervasive in captivity such as artificial diets, sterilized built environments, human interaction, and medical intervention [10–12] may cause changes to the microbiome, but such changes remain undescribed.

Altering microbial communities may have costs to host health as microbes continue to be recognized for their roles in immune function, pathogen defense, and digestion [13]. Coccidiosis, a gut parasite caused by protozoan *Eimeria spp.*, is a common disease in captive kiwi [14]. However, the relationship between captivity, coccidia, and gut microbial communities has garnered little attention. We sought to compare gut bacteria and fungi between captive and wild kiwi. We tested the hypothesis that captivity status and history of coccidiosis would decrease diversity and modify composition of the gut microbiome.

## Results

**Fig. 1:**
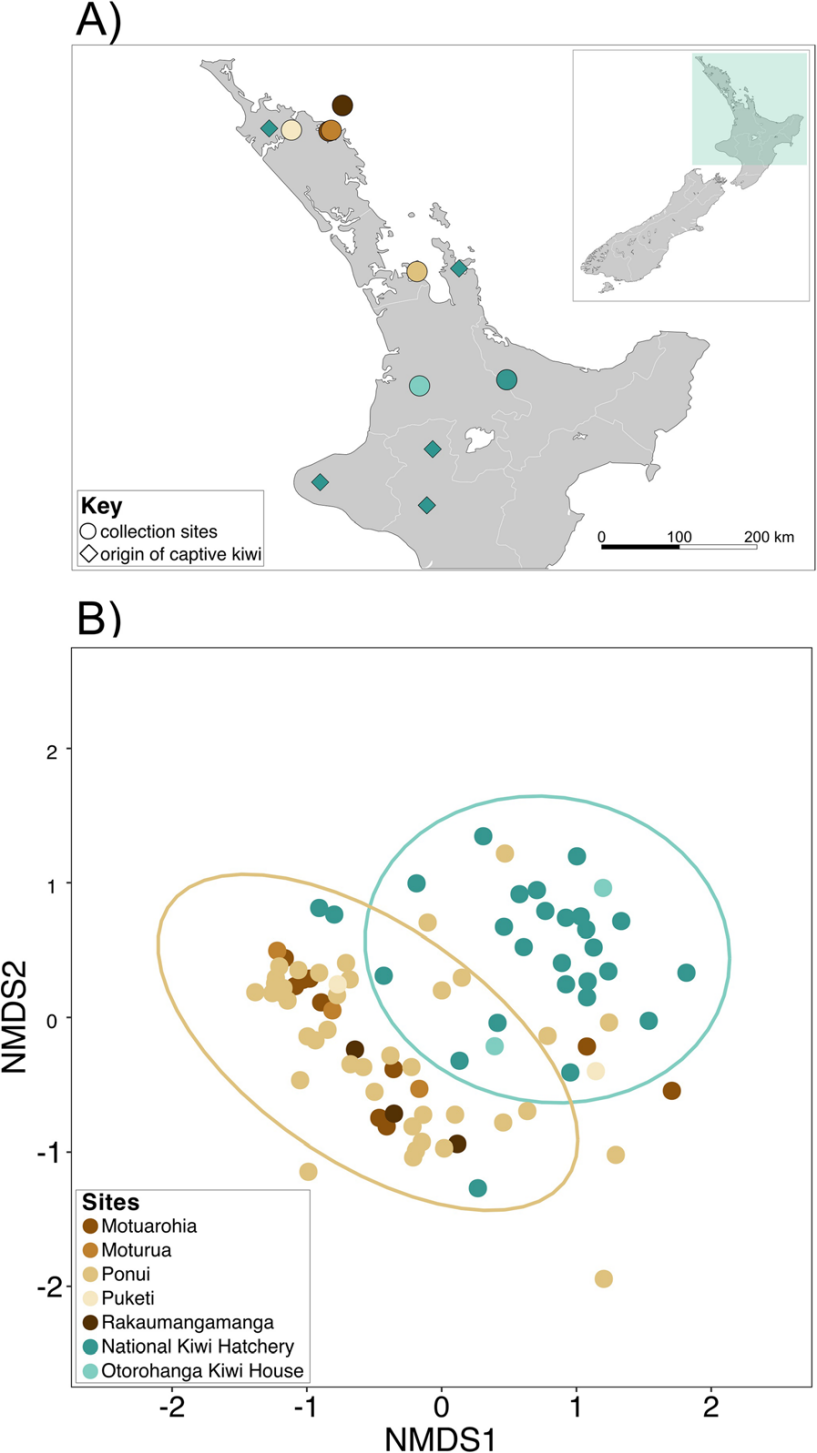
Captivity but not geography shifts the Brown Kiwi gut bacterial composition. **A** Map of collection sites and captive kiwi origin sites where shapes indicate type of site. Collection sites are locations where kiwi feces were sampled and origin sites are where kiwi eggs were lifted. Colors correspond to key in panel **b**. Teal colors correspond to captive sites, while brown colors correspond to wild sites. **B** NMDS plot using Bray-Curtis distance metric shows samples clustering by captivity status with little overlap between the groups (PERMANOVA, *r*^*2*^ = 0.07, *p* = 0.001). Ellipses denote 95 % confidence level

Fresh fecal samples were collected from seven sites on the north island of New Zealand (Fig. 1a, Supplementary Table 1) during January – April 2019.Bacterial 16 S rRNA (V4 region) [15] and fungal ITS genes [16] were amplified using DNA extracted from captive (*n* = 38) and wild kiwi fecal samples (*n* = 68). To ensure our findings were not an artefact of spatial autocorrelation, we conducted a Mantel test and found a weak association between the sites and the kiwi gut microbiome, however, it was not statistically significant (Mantel correlation, *r* = 0.138, *p* =0.308). PERMANOVA results (Supplementary Table 2) found a significant association of site in bacteria (*r*^*2*^ = 0.13, *p* = 0.001) and fungi (*r*^*2*^ = 0.183, *p* =0.001), which shows a relationship due to site but not necessarily due to spatial proximity. Kiwi eggs prior to captive rearing were lifted from five locations (Fig. 1a) that span the range of natural sites, indicating that captive lifestyle is more influential than geographic origin.

**Fig. 2:**
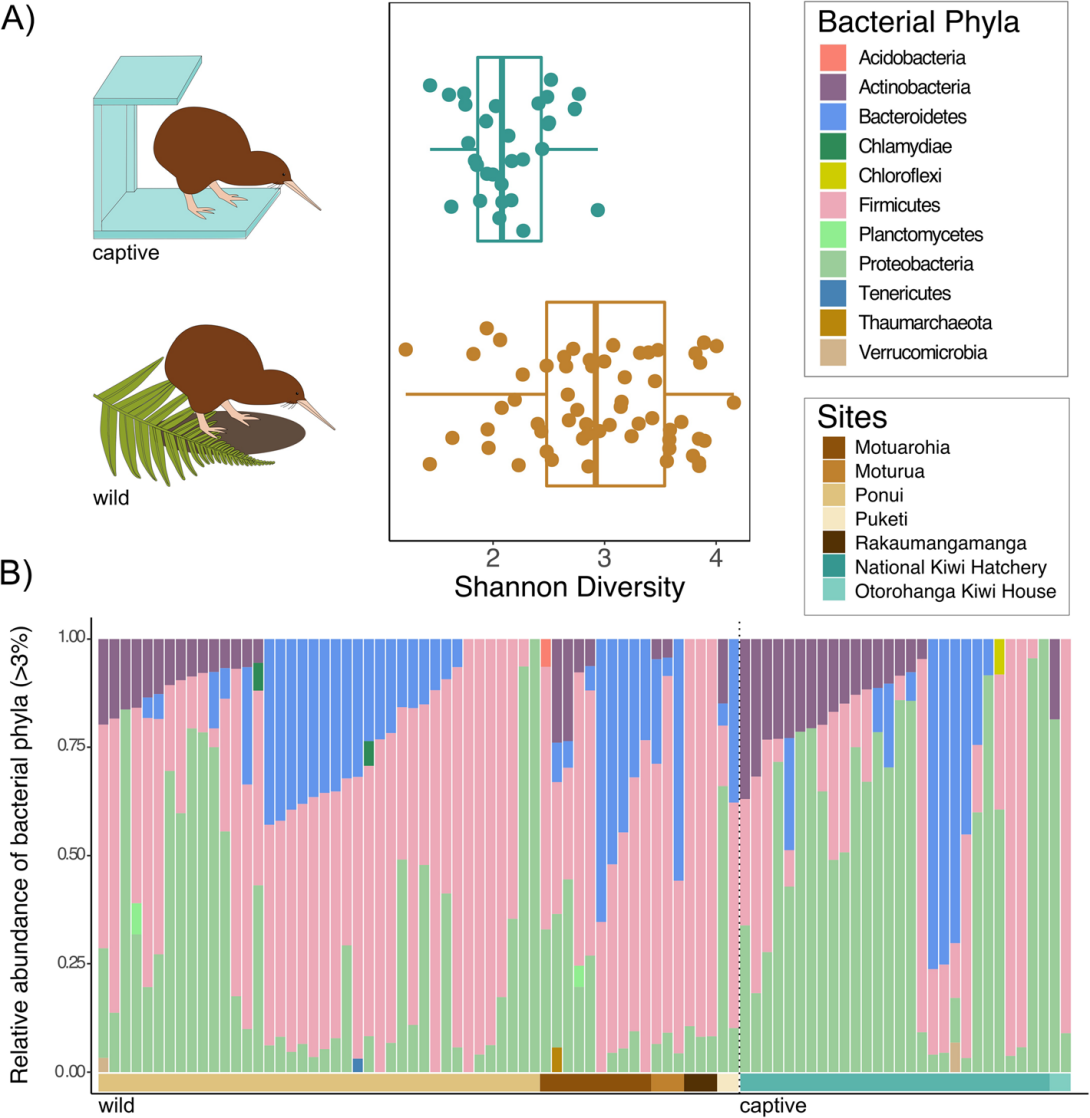
The Brown Kiwi bacterial community differs both in diversity and composition due to captivity status*.* **A** Alpha diversity of captive kiwi is significantly reduced compared to wild individuals (linear model, *r*^*2*^ = 0.288, *p* < 0.001). **B** Relative abundances of bacterial phyla present at > 3 % between captive and wild kiwi. Vertical bars show the bacterial taxa and horizontal bars denote the collection site

To determine whether captivity influences kiwi gut microbiota, we used PERMANOVA, linear models, and NMDS analyses. Bacterial communities clustered by captivity across spatially independent sites with little overlap between the 95 % confidence interval ellipses (Fig. 1b, PERMANOVA, *r*^*2*^ = 0.07, *p* = 0.001). We fitted separate linear models for bacteria and fungi, using log transformed principal coordinates axis 1 that explained 12.7 % of bacterial variation and 12.8 % of fungal variation, as a proxy for community composition, as our response variables. We found that captivity was a significant predictor of bacterial composition (*r*^*2*^ = 0.30, *p* < 0.001) but not fungal composition (*r*^*2*^ = 0.003, *p* = 0.301). Although bacterial phyla composition was variable within and across captivity status, Firmicutes was more prevalent in wild kiwi, while Proteobacteria dominated captive kiwi (Fig. 2b). Three fungal phyla, Ascomycota, Basidiomycota, and Mucoromycota, which contained nine classes were predominant in kiwi feces, and varied within and across captivity status (Supplementary Fig. 3).

Bacterial (Fig. 2a, ANOVA, *p* < 0.005) and fungal (Supplementary Fig. 1, ANOVA, *p* = 0.012) alpha diversity were significantly lower in captive kiwi by 33 and 74 % respectively. Using Shannon diversity (alpha diversity) as a response variable, we fitted a linear model to determine the relationship with captivity status and found captivity to be a significant predictor of bacterial (*r*^*2*^ = 0.288, *p* < 0.001) and fungal (*r*^*2*^ = 0.135, *p* = 0.012) alpha diversity. To assess the spread of variation among kiwi microbiomes in captive and wild conditions, we calculated distance to centroid, a metric for beta diversity. No discernible pattern was observed for bacteria (Supplementary Fig. 2a, ANOVA, *p* = 0.948), but a marginally significant pattern was detected in fungal communities where the mean value was higher in captive kiwi (Supplementary Fig. 2b, ANOVA, *p* = 0.051).

We also tested if site (a factor nested within captivity status) and history of coccidiosis (positive or negative) had an influence on variation in microbial communities using PERMANOVA (Supplementary Table 2). Site showed a significant effect on bacteria (*r*^*2*^ = 0.129, *p* = 0.001) and fungi (*r*^*2*^ = 0.183, *p* = 0.001). We ran a linear model using log transformed principal coordinates axis 1 as a proxy for community composition to test the influence of coccidiosis history, data only available for captive samples, on gut microbiota. We found a significant trend with bacteria (Supplementary Fig. 4, linear model, *r*^*2*^ = 0.118, *p* = 0.041) but not fungi (*r*^*2*^ = 0.043, *p* = 0.204). This contested our PERMANOVA findings which found a weak relationship that was not significant in bacteria (*r*^*2*^ = 0.048, *p* = 0.095) and fungi (*r*^*2*^ = 0.074, *p* = 0.087).

**Fig. 3:**
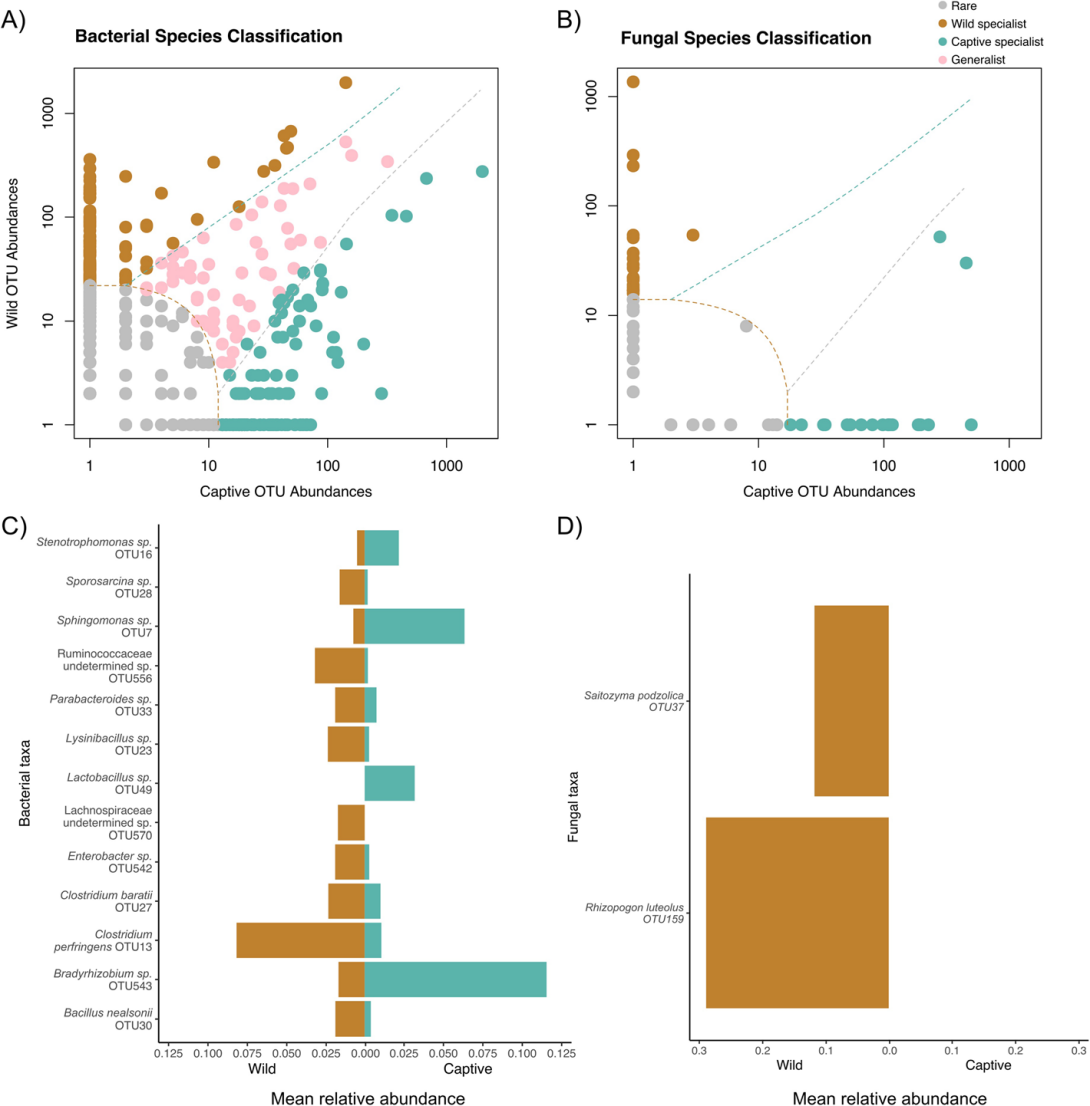
Distinct microbial taxa are classed by their representation in captive and wild kiwi*.* A multinomial species classification method (clamtest) categorized bacterial and fungal OTUs into one of four classes: rare, generalist, wild specialist, and captive specialist. **A** For bacterial OTUs, 9.9 % were classed as generalist, 53 % as rare, 19.7 % as wild specialist, and 17.4 % as captive specialist. **B** For fungal OTUs, 0 % were classed as generalist, 46.7 % as rare, 26.7 % as wild specialist, and 26.7 % as captive specialist. Simper analysis detected several OTUs that explained 70 % difference between captive and wild kiwi. OTUs that were classed as either wild specialist or captive specialist in the clamtest were also represented in the same condition with simper. **C** Nine bacterial OTUs were significantly represented in wild kiwi and four bacterial OTUs in captive kiwi (FDR adjusted *p* < 0.05). **D** Two fungal OTUs were significantly represented in wild kiwi (FDR adjusted *p* < 0.05)

Using a multinomial species classification method (clamtest) [17], we categorized OTUs into four classes: rare, generalist, wild specialist, and captive specialist. For bacterial OTUs, 10 % were classed as generalist, 53 % as rare, 20 % as wild specialist, and 17 % as captive specialist (Fig. 3a, Supplementary Table 3). For fungal OTUs, 0 % were classed as generalist, 47 % as rare, 27 % as wild specialist, and 27 % as captive specialist (Fig. 3b, Supplementary Table 3). We conducted a simper analysis [17, 18] to determine the most influential OTUs that differentiate captive and wild kiwi samples for both bacteria and fungi. Thirteen bacterial OTUs and two fungal OTUs accounted for about 70 % of the differences between wild and captive samples (Supplementary Tables 4 and 5). Nine bacterial OTUs were more abundant in wild samples and five OTUs in captive kiwi (Fig. 3c). Two fungal OTUs were abundant only in wild kiwi (Fig. 3d).

## Discussion

Our results indicate that captivity explains bacterial and fungal community differences in the Brown Kiwi gut. Bacterial composition clustered by captivity (Fig. 1b), suggesting that kiwi from the wild are more similar to each other than their captive counterparts, even across geographically distinct sites. Bacterial and fungal alpha diversity were significantly lower in captive kiwi (Fig. 2a, Supplementary Fig. 1). The consequences of reduced microbial diversity between wild and captive kiwi remain unclear, but several studies have linked dysbiosis to higher disease prevalence in a variety of animals, including ostriches and chickens [19, 20]. Coccidiosis history, data only available for captive samples, showed a marginally significant effect (Supplementary Fig. 4). However, these results may be affected by small sample size. Our results suggest a potential link between differences in the microbiome to disease states that requires further exploration. Overall, our results suggest captivity simplifies the kiwi gut microbiome.

The shift in dominant bacterial phyla, Firmicutes to Proteobacteria, from wild to captive samples may be caused by microbially depauperate captive facilities, antibiotic treatment and post hoc probiotic supplementation. Frequent surface disinfection [11] and probiotic treatment [21] have been shown to increase *Proteobacteria* in human subjects. *Lactobacillus* (OTU 49), a common genus in probiotics, was grouped as a captive specialist and is overrepresented in captive kiwi (Fig. 3c). Other captive-associated taxa include *Corynebacterium* (OTU 62), which has been found in the cloaca of penguins and the preen gland of turkeys [22], and *Bacteroides* (OTU 544), normally found in animal hosts but can include potential pathogens [23]. Wild taxa such as, Ruminococcaceae (OTU 556) and Lachnospiraceae (OTU 570) (Fig. 3c), were overrepresented in wild kiwi. These two bacterial families were found in broiler chickens challenged with *Clostridium perfringens* [24], also a predominant wild taxa (OTU 13), suggesting a relationship between these taxa. *Blautia* (OTU 290), also common in wild kiwi, is a genus found in the human gut and associated with visceral fat accumulation [25]. *Faecalitalea cylindroides* (OTU 687), a butryrate producing microbe, has been detected in chicken [26]. These taxa may be indicative of nutrient acquisition in the wild, where food may be less available.

No fungal OTUs were categorized as generalists suggesting fungi in kiwi reflect their local environment. Some captive specialists include *Cladosporium* (OTU 151) and *Aureobasidium* (OTU 2), both associated with indoor environments and plant material [27, 28], implicating the contribution of soil and ferns added to enclosures. *Trichosporon* (OTU 171), another captive specialist, is a common human skin taxa [29], suggesting close human interaction may shape kiwi fungi. One wild specialist that is abundant in wild samples (Fig. 3d), *Rhizopogon luteolus* (OTU 159), has been identified as a dietary component of small mammals, suggesting kiwi may be consuming and dispersing these fungi [30]. *Preussia* (OTU 181) and *Saitozyma podzolica* (OTU 37), both associated with soil and litter, were grouped as wild specialists [31, 32].

## Conclusions

In captivity, artificial diet, sterilized built environments, and human interaction are key factors that can shape gut microbial communities [10, 11]. Further detailed investigation of how gut microbes establish in developing kiwi chicks can elucidate how these factors inherent to captivity contribute to the kiwi gut microbiome. Overall, our data suggest that captivity explains differences in the gut microbiome of the Brown Kiwi with potential for health and disease assessment for captive-reared individuals.

## Methods

### Study system

#### Captive

Samples were collected (*n* = 38) from two captive sites (Fig. 1a). The National Kiwi Hatchery is located at the Rainbow Springs Nature Park in Rotorua, New Zealand. It is the leading facility in kiwi husbandry, egg incubation, and kiwi rearing. The facility has hatched and reared nearly 2000 kiwi eggs. Otorohanga Kiwi House is located in Otorohanga, New Zealand. Both facilities are a part of the Operation Nest Egg (ONE), a program in which kiwi eggs laid in the wild are transported to a hatchery and reared in a captive environment. Coccidiosis information was only available for the captive samples, where diagnosis is determined using a fecal flotation assay [33]. Kiwi were housed in brooder boxes – wooden boxes with soil, food, and water. Captive kiwi eat a diet mainly consisting of ox heart, cat biscuits, and rolled oats [34]. If positive for parasites or infections, individuals are administered antiprotozoal or antibiotic treatment. After antibiotics, kiwi are given probiotics that include a combination of *Lactobacillus spp.* and *Bifidobacterium lactis*.

#### Wild

Samples were collected (*n* = 68) from five natural sites with established wild kiwi populations (Fig. 1a). Ponui Island is located 30 km east of Auckland, New Zealand. 14 Brown Kiwi were introduced to the island by the New Zealand Wildlife Service in 1964, where populations have been increasing, establishing one of the densest populations of kiwi at an estimated 1500 individuals. Motuarohia Island is located in the Bay of Islands, 4 km northeast of Russell, New Zealand. Moturua Island is east of Motuarohia in the Bay of Islands. Puketi Forest is located in the Northland region of New Zealand. Rakaumangamanga is located near the Bay of Islands. These sites differ in climate and vegetation [8] and home to a number of Brown Kiwi individuals.

### Sample collection

Fresh fecal samples (*n* = 108) were collected using sterile spatulas. Supplementary Table 1 details the quantity of samples collected per site. The interior of the fecal pellet was collected to ensure minimal environmental exposure. Due to the unique scent of kiwi excreta, we used the sample’s scent to confirm the feces was of kiwi origin [35]. Fecal samples were stored in 5 mL Eppendorf tubes suspended in molecular grade (100 %) ethanol and stored in -20ºC. DNA was extracted using MN NucleoSpin Soil Kit (Macherey-Nagel, Duren, Germany) on Janus extraction robot (PerkinElmer, Waltham, United States), suspended in TE buffer, and stored in -20ºC until PCR amplification.

### Metabarcoding

We used a metabarcoding approach with a two-stage amplification process. During the first stage, we amplified the V4 region of the bacterial 16 S rRNA gene using 515 F/806R primers [15] and the fungal ITS gene [16]. The following PCR parameters were applied: denaturation at 95ºC for 2 min, followed by 35 cycles at 95ºC for 20 s, 50ºC for 20 s, and at 72ºC for 30 s, and final extension at 72ºC for 1 min. We used the resulting PCR products as template DNA in the second-stage PCR. Barcoded Fusion primers were used with the following PCR parameters: initial denaturation at 95ºC for 2 min, followed by 8 cycles of 95ºC for 20 s, 50ºC for 20 s, and 72ºC for 50 s, and final extension at 72ºC for 10 min [15]. We purified the second-stage PCR products using SeraMag magnetic beads to remove primer dimers and normalize concentration [36]. Qubit (dsDNA HS Assay Kit, Invitrogen, Carlsbad, United States) was used to quantify DNA concentration and libraries were diluted to 4 nM prior to final pooling. We pooled the libraries according to the concentration determined by Qubit, equimolar based on number of samples per library, and amplicon length. We used LabChip GX Touch Nucleic Acid Analyzer (PerkinElmer, Waltham, United States) to determine DNA concentration and assess quality of final pooled library. Samples were sequenced using Illumina MiSeq platform at Auckland Genomics Facility (University of Auckland), phiX spike 10 %, 250 × 2 cycles. Bioinformatics pipeline Claident was used to demultiplex raw sequences [37]. PEAR evaluated all possible paired-end read overlaps and merged sequences [38]. VSEARCH filtered noisy reads, removed chimeras, and clustered sequences into operational taxonomic units (OTUs) [39]. Claident clustered sequences into OTUs at 97 % similarity and assigned taxonomy with RDP classifier using the following databases, 16 S rRNA training set 16 (bacteria) and UNITE fungal ITS train set 07-04-2014 (fungi). Bioinformatic analysis were performed on the NeSI HPC environment.

### Statistical analysis

We calculated Shannon diversity index (R package *phyloseq* version 2.5-7) [40] to test for a relationship between microbial alpha diversity and captivity. We calculated beta diversity using a multivariate version of Levene’s test for homogeneity of variances (betadisper in R package *vegan* version 2.5-7) [17]. We reported the distance to centroid value. To test for spatial autocorrelation among sites, we conducted a Mantel test (R package *ade4* version 1.7–16) using microbiome data, and site location data (latitude and longitude) [41].

We used non-metric multidimensional scaling (NMDS) with Bray-Curtis dissimilarity matrices to reduce multivariate data and spatially visualize microbial communities. NMDS was used to visualize clustering trends across captivity status. We used permutational analysis of the variance (PERMANOVA) also with Bray-Curtis distance matrices to determine whether different factors, such as captivity status (wild/captive), site (geographic area), microsite (i.e. in brooder box, soil, etc.), age (days old of captive individuals), weight (mass in grams for captive individuals), collection date, and history of coccidiosis (positive/negative) can explain microbial community variance. In addition, we ran linear models to determine whether captivity status or coccidiosis history were good predictors of both bacterial and fungal alpha diversity and community composition. We used Shannon Diversity Index values as a response variable for alpha diversity. In our community composition models, we used principal coordinates analysis axis values as our response variable. To fit the assumptions of the model and accommodate negative values, we added the minimum value plus one and log transformed the data. We ran a mixed effects model using site as a random effect, however, Akaike information criterion (AIC) and ANOVA confirmed that it did not improve the fit.

We used clamtest [17] to categorize bacterial and fungal OTUs into the following groups: generalist, too rare, and group specialist (wild-, captive-, positive-, negative-). Positive and negative correspond to individual kiwi who have had a history of coccidiosis. We conducted a simper analysis [17, 18] to determine which OTUs explain over 70 % of the differences between groups and to identify OTUs that are overrepresented.

## Supplementary Information

**Additional file 1: Supplementary Figure 1. **Fungal alpha diversity significantly decreases from wild to captive. Using Shannon’s diversity index, there is a 74.2% reduction in the average alpha diversity (ANOVA, *p* = 0.012) (linear model, *r*^*2*^ = 0.1348, *p* = 0.01233).

**Additional file 2: Supplementary Figure 2. **Betadiversity of bacterial and fungal communities vary in their response to captivity. (A) There is no significant difference in betadiversity (distance to centroid) observed in bacteria (ANOVA, *p* = 0.948). (B) Fungal betadiversity shows a marginally significant trend with an increase in distance to centroid (ANOVA, *p* = 0.051). Distance to centroid was calculated using a multivariate version of the Levene’s test. Lower values indicate more shared microbial taxa among individuals of the same treatment. Higher values show higher microbial taxa variability among individuals of the same treatment.

**Additional file 3: Supplementary Figure 3. **The Brown Kiwi fungal community is highly variable within and across groups. Relative abundances of fungi classes present at > 3% between captive and wild kiwi.

**Additional file 4: Supplementary Figure 4. **History of coccidiosis influences kiwi gut bacteria. PCoA plot using Bray-Curtis distance metric shows samples clustering by coccidiosis history (PERMANOVA, *r*^*2*^ = 0.048, *p* = 0.095)(linear model, *r*^*2*^ = 0.1183, *p*= 0.041). Ellipses denote 95% confidence level.

**Additional file 5: Supplementary Table 1. ** Sample collection sites along with the latitude and longitude, captivity status, and sampling size.

**Additional file 6: Supplementary Table 2. **Captivity influences bacterial and fungal communities. PERMANOVA results for different factors as predictors of microbial variance. Number of asterisks indicate level of statistical significance (***p < 0.001, **p < 0.01, *p < 0.05).

**Additional file 7: Supplementary Table 3. ** Clamtest categorizing bacterial and fungal OTUs found in wild and captive kiwi into rare, generalist, wild specialist, and captive specialist.

**Additional file 8: Supplementary Table 4. ** Most influential bacterial OTUs distinguishing between wild and captive kiwi samples listed by highest contributing OTU in descending order. Thirteen bacterial OTUs significantly account for over 70% of the differences between captivity status. OTUs that contributed to less than 1% significance was removed. A p-value was calculated per OTU, in addition to false discovery rate (FDR) adjusted p-value. Mean abundance and standard deviation of each OTU is listed between groups.

**Additional file 9: Supplementary Table 5. ** Most influential fungal OTUs distinguishing between wild and captive kiwi samples listed by highest contributing OTU in descending order. Two fungal OTUs significantly account for over 70% of the differences between captivity status. OTUs that contributed to less than 1% significance were removed. A p-value was calculated per OTU, in addition to false discovery rate (FDR) adjusted p-value. Mean abundance and standard deviation of each OTU is listed between groups.

**Additional file 10: Supplementary Table 6. **Clamtest categorizing bacterial and fungal OTUs found in captive kiwi with and without a history of coccidiosis into rare, generalist, positive specialist, and negative specialist.

**Additional file 11.**

## Data Availability

The datasets generated and/or analyzed during the current study are available in a github repository, https://github.com/psanjuan/kiwi_microbiome_2019.

## Abbreviations

OTU: Operational taxonomic unit
PCR: Polymerase chain reaction
DNA: Deoxyribonucleic nucleic acid
rRNA: Ribosomal ribonucleic acid
PERMANOVA: Permutational analysis of the variance
NMDS: Non-metric multidimensional scaling
