# Supplementary figures and images for: Captivity reduces diversity and shifts composition of the Brown Kiwi microbiome
Source: Anim Microbiome. 2021 Jul 8;3:48. doi: 10.1186/s42523-021-00109-0 (PMC8268595; doi:10.1186/s42523-021-00109-0)

Fungal alpha diversity

Shannon Diversity

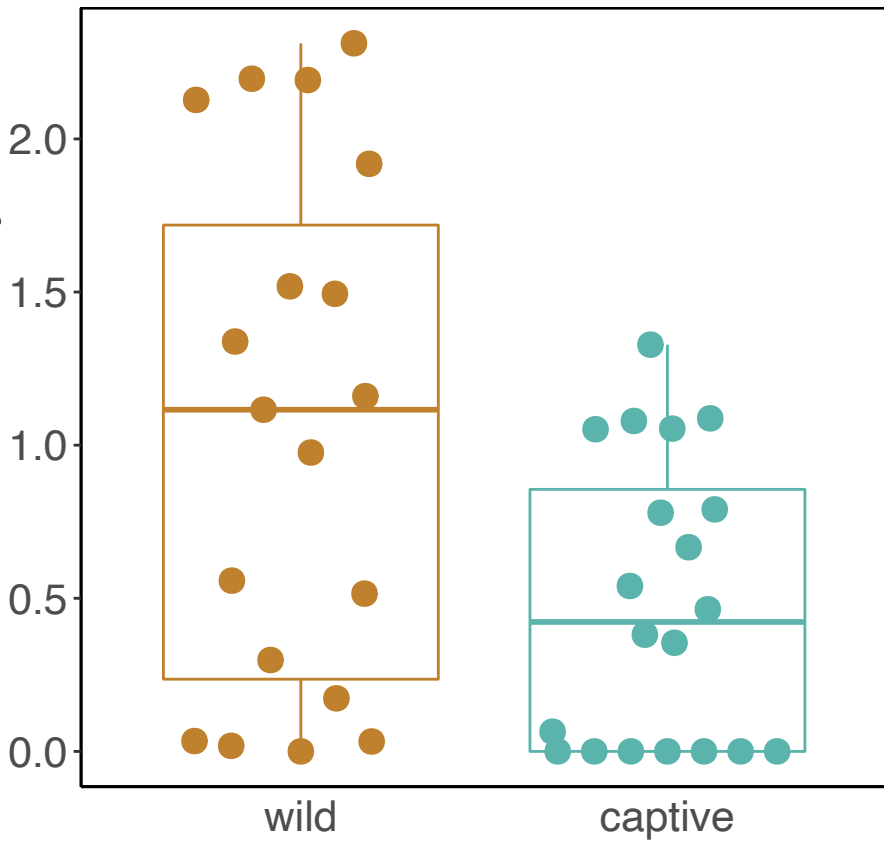

Supplement: Supplementary file 1 — Additional file 1: Supplementary Figure 1. Fungal alpha diversity significantly decreases from wild to captive. Using Shannon’s diversity index, there is a 74.2% reduction in the average alpha diversity (ANOVA, p = 0.012) (linear model, r2 = 0.1348, p = 0.01233). [file 42523_2021_109_MOESM1_ESM.pdf]

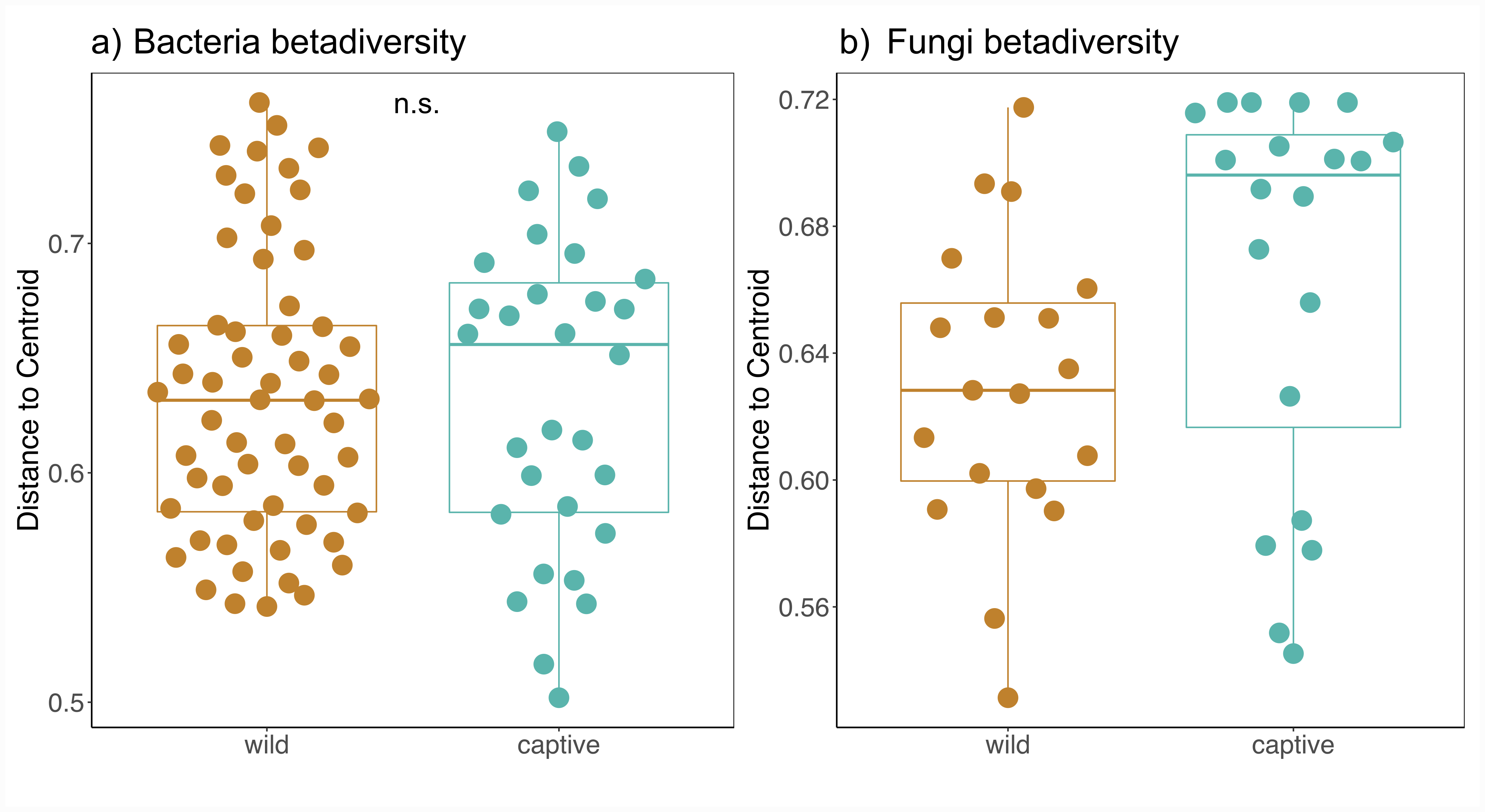

Supplement: Supplementary file 2 — Additional file 2: Supplementary Figure 2. Betadiversity of bacterial and fungal communities vary in their response to captivity. (A) There is no significant difference in betadiversity (distance to centroid) observed in bacteria (ANOVA, p = 0.948). (B) Fungal betadiversity shows a marginally significant trend with an increase in distance to centroid (ANOVA, p = 0.051). Distance to centroid was calculated using a multivariate version of the Levene’s test. Lower values indicate more shared microbial taxa among individuals of the same treatment. Higher values show higher microbial taxa variability among individuals of the same treatment. [file 42523_2021_109_MOESM2_ESM.tiff]

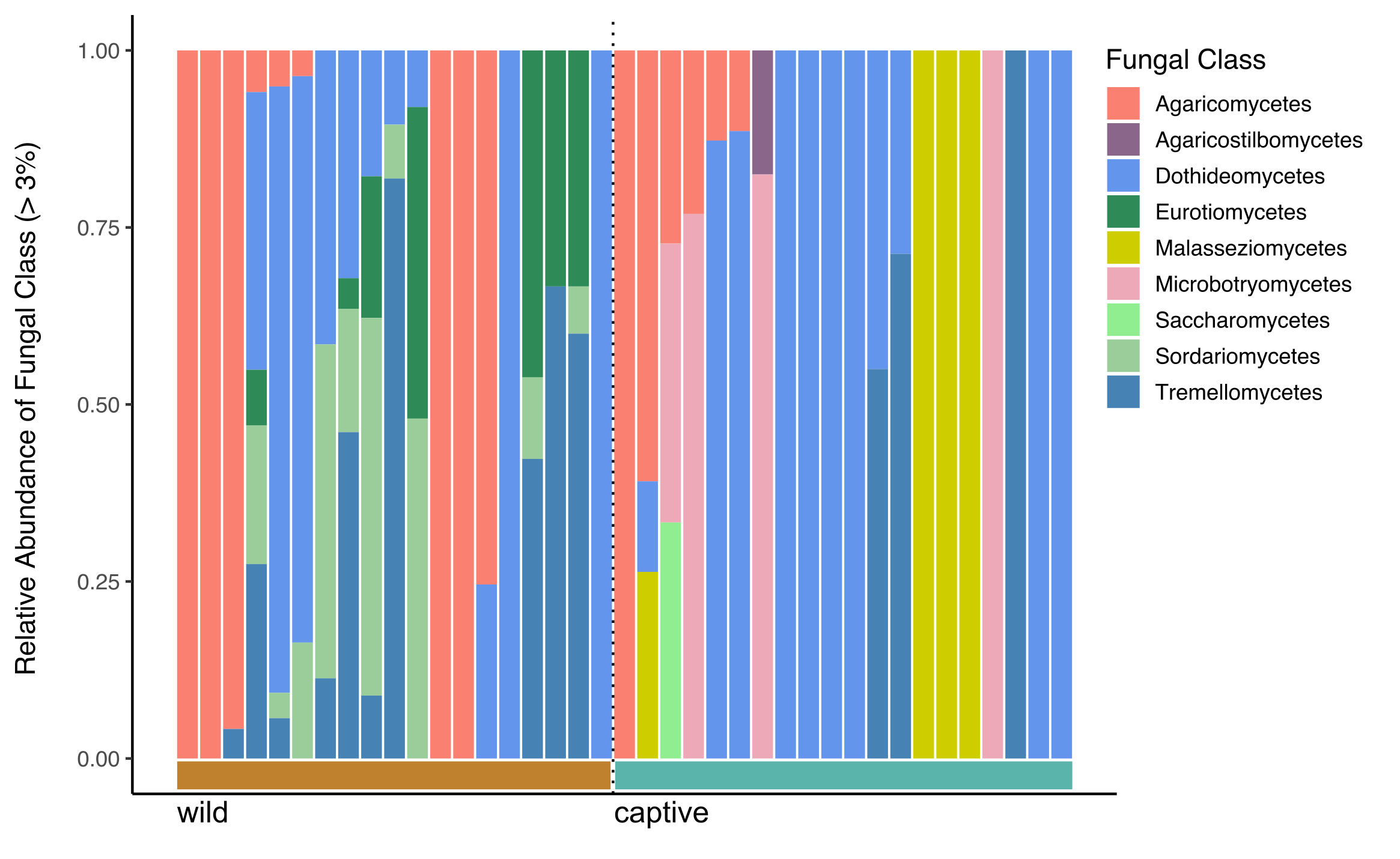

Supplement: Supplementary file 3 — Additional file 3: Supplementary Figure 3. The Brown Kiwi fungal community is highly variable within and across groups. Relative abundances of fungi classes present at > 3% between captive and wild kiwi. [file 42523_2021_109_MOESM3_ESM.tiff]

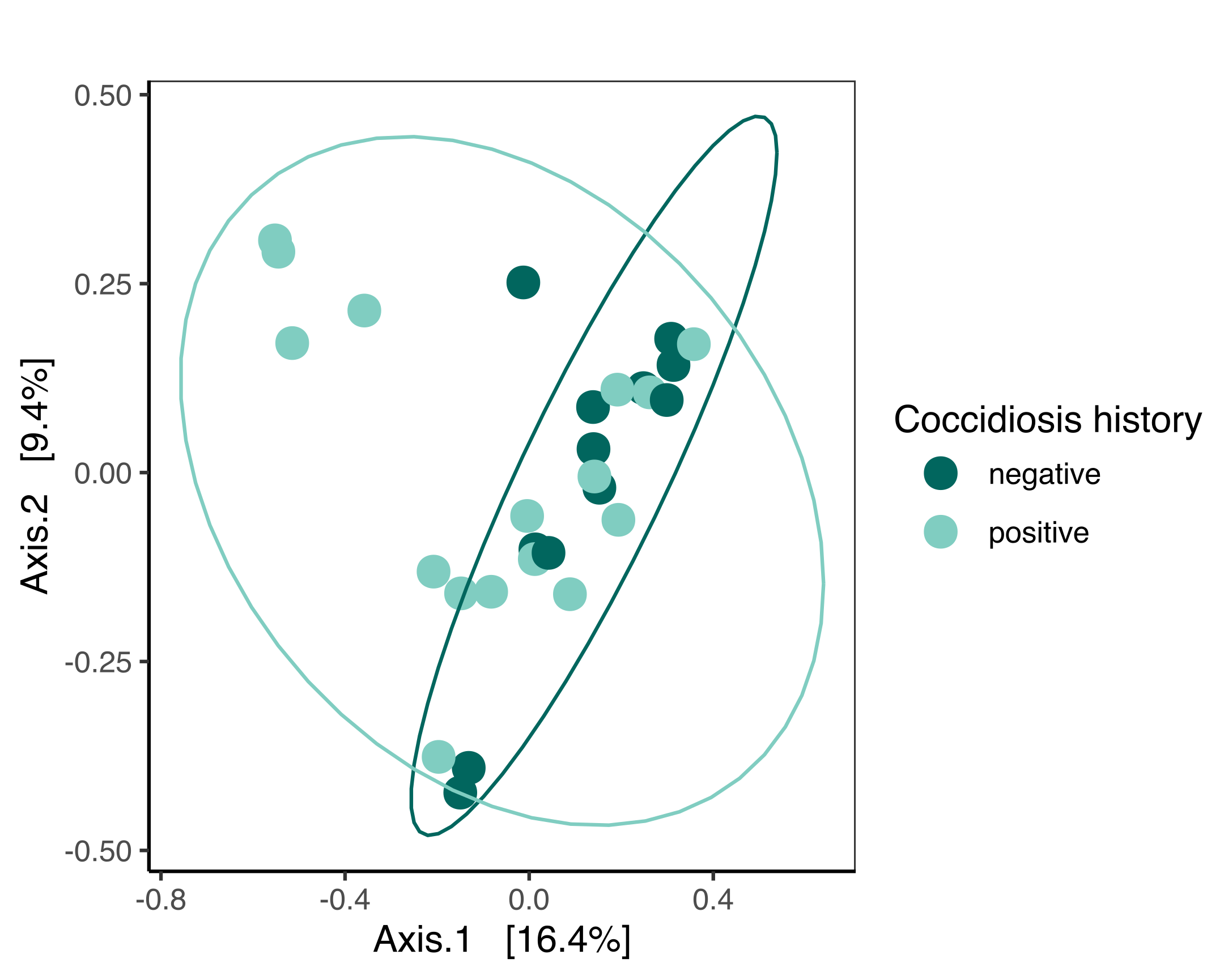

Supplement: Supplementary file 4 — Additional file 4: Supplementary Figure 4. History of coccidiosis influences kiwi gut bacteria. PCoA plot using Bray-Curtis distance metric shows samples clustering by coccidiosis history (PERMANOVA, r2 = 0.048, p = 0.095)(linear model, r2 = 0.1183, p= 0.041). Ellipses denote 95% confidence level. [file 42523_2021_109_MOESM4_ESM.tiff]
