# Supplementary material for: Captivity reduces diversity and shifts composition of the Brown Kiwi microbiome
Source: Anim Microbiome. 2021 Jul 8;3:48. doi: 10.1186/s42523-021-00109-0 (PMC8268595; doi:10.1186/s42523-021-00109-0)
Supplement: Supplementary file 5 — Additional file 5: Supplementary Table 1. Sample collection sites along with the latitude and longitude, captivity status, and sampling size. [file 42523_2021_109_MOESM5_ESM.pdf]

Supplementary Table 1: Sample collection sites along with the latitude and longitude, captivity status, and sampling size

Site name	Latitude	Longitude	Captivity status	Sample size
Motuarohia	-35.23184	174.165382	wild	15
Moturua	-35.221929	174.192224	wild	3
Ponui	-36.859947	175.184151	wild	44
Puketi	-35.219586	173.732208	wild	3
Rakaumangamanga	-34.937289	174.322347	wild	3
National Kiwi Hatchery	-38.108791	176.221903	captive	35
Otorohanga Kiwi House	-38.179541	175.213373	captive	3
**TOTAL:**				106
