# Supplementary material for: Captivity reduces diversity and shifts composition of the Brown Kiwi microbiome
Source: Anim Microbiome. 2021 Jul 8;3:48. doi: 10.1186/s42523-021-00109-0 (PMC8268595; doi:10.1186/s42523-021-00109-0)
Supplement: Supplementary file 6 — Additional file 6: Supplementary Table 2. Captivity influences bacterial and fungal communities. PERMANOVA results for different factors as predictors of microbial variance. Number of asterisks indicate level of statistical significance (***p < 0.001, **p < 0.01, *p < 0.05). [file 42523_2021_109_MOESM6_ESM.pdf]

Supplementary Table 2: Captivity influences bacterial and fungal communities. PERMANOVA results for different factors as predictors of microbial variance. Number of asterisks indicate level of statistical significance (***p < 0.001, **p < 0.01, *p < 0.05).

Taxa	Factor	PERMANOVA		
		r^2	p-value	
Bacteria	captivity status (captive or wild)	0.07	0.001	***
	collection site	0.129	0.001	***
	microsite	0.407	0.001	***
	coccidiosis history (positive or negative)	0.048	0.095	
	weight	0.056	0.018	*
	age	0.054	0.805	
	collection date	0.0307	0.001	***
Fungi	captivity status	0.073	0.001	***
	collection site	0.183	0.001	***
	microsite	0.301	0.023	*
	coccidiosis history (positive or negative)	0.074	0.087	
	weight	0.059	0.438	
	age	0.205	0.4	
	collection date	0.03997	0.05	*
