# Supplementary material for: Captivity reduces diversity and shifts composition of the Brown Kiwi microbiome
Source: Anim Microbiome. 2021 Jul 8;3:48. doi: 10.1186/s42523-021-00109-0 (PMC8268595; doi:10.1186/s42523-021-00109-0)
Supplement: Supplementary file 7 — Additional file 7: Supplementary Table 3. Clamtest categorizing bacterial and fungal OTUs found in wild and captive kiwi into rare, generalist, wild specialist, and captive specialist. [file 42523_2021_109_MOESM7_ESM.pdf]

Supplementary Table 3: Clamtest categorizing bacterial and fungal OTUs found in wild and captive kiwi into rare, generalist, wild specialist, and captive specialist.

**Taxa**	**Category**	**Number of OTUs**	**Proportion**
Bacteria	Generalist	58	0.099
	Wild specialist	115	0.197
	Captive specialist	102	0.174
	Rare	310	0.53
Fungi	Generalist	0	0
	Wild specialist	20	0.267
	Captive specialist	20	0.267
	Rare	35	0.467
