# Supplementary material for: Captivity reduces diversity and shifts composition of the Brown Kiwi microbiome
Source: Anim Microbiome. 2021 Jul 8;3:48. doi: 10.1186/s42523-021-00109-0 (PMC8268595; doi:10.1186/s42523-021-00109-0)
Supplement: Supplementary file 8 — Additional file 8: Supplementary Table 4. Most influential bacterial OTUs distinguishing between wild and captive kiwi samples listed by highest contributing OTU in descending order. Thirteen bacterial OTUs significantly account for over 70% of the differences between captivity status. OTUs that contributed to less than 1% significance was removed. A p-value was calculated per OTU, in addition to false discovery rate (FDR) adjusted p-value. Mean abundance and standard deviation of each OTU is listed between groups. [file 42523_2021_109_MOESM8_ESM.pdf]

Supplementary Table 4: Most influential bacterial OTUs distinguishing between wild and captive kiwi samples listed by highest contributing OTU in descending order. 13 bacterial OTUs significantly account for over 70% of the differences between captivity status. OTUs that contributed to less than 1% significance was removed. A p-value was calculated per OTU, in addition to false discovery rate (FDR) adjusted p-value. Mean abundance and standard deviation of each OTU is listed between groups.

OTU	Taxonomic level	Taxa	Percent total contribution	p-value	FDR adjusted p-value	Wild mean abundance	Wild standard deviation	Captive mean abundance	Captive standard deviation
OTU13	species	*Clostridium perfringens*	0.053655621	0.000136263	0.000772157	0.081879789	0.140470551	0.010099208	0.051453687
OTU543	genus	*Bradyrhizobium sp.*	0.045988157	7.76E-06	0.000131945	0.01718544	0.032971642	0.115425213	0.13622254
OTU7	genus	*Sphingomonas sp.*	0.025692136	5.65E-05	0.000480146	0.007784795	0.026203229	0.063389749	0.103807902
OTU27	species	*Clostridium baratii*	0.02001042	0.025545768	0.039479823	0.023623748	0.089747899	0.010609481	0.05811052
OTU556	family	Ruminococcaceae	0.019127641	0.000357966	0.001217084	0.032128992	0.053891864	0.002083422	0.007431549
OTU49	genus	*Lactobacillus sp.*	0.016133391	0.000724079	0.002051556	1.91E-05	0.000145411	0.031783684	0.098714368
OTU23	genus	*Lysinibacillus sp.*	0.015537456	0.009301912	0.017570278	0.02395652	0.066973113	0.002835537	0.011701813
OTU33	genus	*Parabacteroides sp.*	0.014370846	0.008179659	0.017381775	0.019284762	0.036641955	0.007530305	0.021620192
OTU30	species	*Bacillus nealsonii*	0.011491221	0.010420885	0.017715505	0.019156704	0.048826459	0.003954601	0.021397974
OTU28	genus	*Sporosarcina sp.*	0.011278153	0.030220208	0.040914297	0.016472807	0.058383151	0.001932169	0.009238382
OTU542	genus	*Enterobacter sp.*	0.011133118	0.002137812	0.005191829	0.019289558	0.059545879	0.002913295	0.014544268
OTU16	genus	*Stenotrophomonas sp.*	0.011076259	0.031287404	0.040914297	0.005396551	0.02443941	0.021684426	0.052999438
OTU570	family	Lachnospiraceae	0.010361122	0.000319224	0.001217084	0.01756036	0.04886533	0	0
