# Supplementary material for: Captivity reduces diversity and shifts composition of the Brown Kiwi microbiome
Source: Anim Microbiome. 2021 Jul 8;3:48. doi: 10.1186/s42523-021-00109-0 (PMC8268595; doi:10.1186/s42523-021-00109-0)
Supplement: Supplementary file 9 — Additional file 9: Supplementary Table 5. Most influential fungal OTUs distinguishing between wild and captive kiwi samples listed by highest contributing OTU in descending order. Two fungal OTUs significantly account for over 70% of the differences between captivity status. OTUs that contributed to less than 1% significance were removed. A p-value was calculated per OTU, in addition to false discovery rate (FDR) adjusted p-value. Mean abundance and standard deviation of each OTU is listed between groups. [file 42523_2021_109_MOESM9_ESM.pdf]

Supplementary Table 5: Most influential fungal OTUs distinguishing between wild and captive kiwi samples listed by highest contributing OTU in descending order. Two fungal OTUs significantly account for over 70% of the differences between captivity status. OTUs that contributed to less than 1% significance were removed. A p-value was calculated per OTU, in addition to false discovery rate (FDR) adjusted p-value. Mean abundance and standard deviation of each OTU is listed between groups.

OTU	Taxonomic level	Taxa	Percent total contribution	p-value	FDR adjusted p-value	Wild mean abundance	Wild standard deviation	Captive mean abundance	Captive standard deviation
OTU159	genus	*Rhizopogon luteolus*	0.174474915	0.003289986	0.023029899	0.289177443	0.440625708	0	0
OTU37	species	*Saitozyma podzolica*	0.059634377	0.000613432	0.008588045	0.118427688	0.183853323	0	0
