# Supplementary material for: Captivity reduces diversity and shifts composition of the Brown Kiwi microbiome
Source: Anim Microbiome. 2021 Jul 8;3:48. doi: 10.1186/s42523-021-00109-0 (PMC8268595; doi:10.1186/s42523-021-00109-0)
Supplement: Supplementary file 10 — Additional file 10: Supplementary Table 6. Clamtest categorizing bacterial and fungal OTUs found in captive kiwi with and without a history of coccidiosis into rare, generalist, positive specialist, and negative specialist. [file 42523_2021_109_MOESM10_ESM.pdf]

Supplementary Table 6: Clamtest categorizing bacterial and fungal OTUs found in kiwi with and without a history of coccidiosis into rare, generalist, positive specialist, and negative specialist.

Taxa	Category	Number of OTUs	Proportion
Bacteria	Generalist	23	0. 092
	Positive-coccidiosis specialist	38	0. 152
	Negative-coccidiosis specialist	42	0. 168
	Rare	147	0. 588
Fungi	Generalist	2	0.057
	Positive-coccidiosis specialist	10	0.286
	Negative-coccidiosis specialist	8	0.229
	Rare	15	0.429
