# Supplementary material for: Captivity reduces diversity and shifts composition of the Brown Kiwi microbiome
Source: Anim Microbiome. 2021 Jul 8;3:48. doi: 10.1186/s42523-021-00109-0 (PMC8268595; doi:10.1186/s42523-021-00109-0)
Supplement: Supplementary file 11 — Additional file 11. [file 42523_2021_109_MOESM11_ESM.docx]

**Supplementary Information**

Captivity reduces diversity and shifts composition of the Brown Kiwi microbiome

Priscilla A. San Juan^1,2,3^, Isabel Castro^4^, and Manpreet Dhami^1,5^

^1^Co-corresponding authors: 371 Serra Mall, Stanford, California 94305; + 1 562 712 5779; 54 Gerald Street, Lincoln, New Zealand 7608; +64 21 211 2174; [psanjuan@stanford.edu](mailto:psanjuan@stanford.edu) and dhamim@landcareresearch.co.nz

^2^Department of Biology, Stanford University, Stanford, California, USA

^3^Center for Conservation Biology, Stanford University, Stanford, California, USA

^4^Wildlife and Ecology Group, School of Agriculture and Environment, Massey University; Palmerston North, New Zealand

^5^Manaaki Whenua – Landcare Research; Lincoln, New Zealand

**Supplemental Results**

**Results and Discussion**

*Microbial diversity*

Shannon diversity metric was calculated to determine if captivity status impacted microbial species diversity. The mean values, for both bacteria and fungi, were significantly lower for captive kiwi (bacteria: ANOVA, *p* < 0.005; fungi: ANOVA, p = 0.012). Betadiversity, reported as distance to centroid, did not change with captivity for bacteria but increased for fungi values (Supplementary Fig. 2). A bimodal distribution has been observed in the captive treatment for both bacteria and fungi, however the factor that explains it is undetermined.

*Factors that influence the kiwi microbiome*

PERMANOVA results reveal that captivity, site, microsite, weight, and collection date are significant predictors of bacterial variance (Supplementary Table 2). Both microsite and site are nested factors within captivity status. History of coccidiosis showed a marginally significant trend, which can be due to treatment of the parasite and microbiome recovery. For fungi, captivity, site, microbiome, and collection date are significant predictors of community variance (Supplementary Table 2). History of coccidiosis also showed a marginally significant trend. Small sample size could be another factor that influences these observed patterns.

*Coccidiosis history and microbial species classification*

A multinomial species classification test (clamtest) was conducted for both captivity status (Figure 3, Supplementary Table 3) and coccidiosis history (Supplementary Table 6). Findings for the captivity clamtest are reported in the main text. For coccidiosis treatment, bacterial and fungal OTUs were grouped into four categories: generalist, too rare, positive-specialist, and negative-specialist. Bacterial taxa that were categorized as positive-specialist include, *Methylobacterium* (OTU 77), *Parasutterella* (OTU 139), *Bilophila* (OTU 172), and *Clostridium* (OTU 688). *Methylobacterium*, a slow growing and biofilm producing microbe, has been recognized as a human pathogen in immunocompromised patients [1]. *Parasutterella,* a taxa responsible for bile acid maintenance and cholesterol metabolism [2]. *Bilophila*, a genus that may include species like ﻿*Bilophila wadsworthia*. *B. wadsworthia* is a pathogen that causes gut inflammation [3]. *Clostridium* is a genus that may contain pathogenic and commensal species [4]. Coccidiosis may be favoring growth of these taxa, with potential to exacerbate symptoms. Fungal OTUs that have been classed as positive-specialist were predominantly environment and skin-associated, which is consistent with our hypothesis that fungal taxa are transient and reflective of their host’s environment. These taxa include: *Sporobolomyces ruberrimus* (OTU 3), *Mortierella hypsicladia* (OTU 53), and *Malassezia restricta* (OTU 208)*.*

**Supplementary references:**

1. Kovaleva J, Degener JE, Van Der Mei HC. Methylobacterium and its role in health care-associated infection. J Clin Microbiol. 2014;52:1317–21.

2. Ju T, Kong JY, Stothard P, Willing BP. Defining the role of Parasutterella, a previously uncharacterized member of the core gut microbiota. ISME J. 2019;13:1520–34. doi:10.1038/s41396-019-0364-5.

3. Natividad JM, Lamas B, Pham HP, Michel ML, Rainteau D, Bridonneau C, et al. Bilophila wadsworthia aggravates high fat diet induced metabolic dysfunctions in mice. Nat Commun. 2018;9:1–15. doi:10.1038/s41467-018-05249-7.

4. Lopetuso LR, Scaldaferri F, Petito V, Gasbarrini A. Commensal Clostridia: Leading players in the maintenance of gut homeostasis. Gut Pathog. 2013;5:1–8.

**Supplementary Figure Legends**

**Supplementary Figure 1:** *Fungal alpha diversity significantly decreases from wild to captive.* Using Shannon’s diversity index, there is a 74.2% reduction in the average alpha diversity (ANOVA, p = 0.012) (linear model, *r^2^* = 0.1348, *p* = 0.01233)*.*

**Supplementary Figure 2:** *Betadiversity of bacterial and fungal communities vary in their response to captivity*. (A) There is no significant difference in betadiversity (distance to centroid) observed in bacteria (ANOVA, *p* = 0.948). (B) Fungal betadiversity shows a marginally significant trend with an increase in distance to centroid (ANOVA, *p* = 0.051). Distance to centroid was calculated using a multivariate version of the Levene’s test. Lower values indicate more shared microbial taxa among individuals of the same treatment. Higher values show higher microbial taxa variability among individuals of the same treatment.

**Supplementary Figure 3:** *The Brown Kiwi fungal community is highly variable within and across groups.* Relative abundances of fungi classes present at > 3% between captive and wild kiwi.

**Supplementary Figure 4:** *History of coccidiosis influences kiwi gut bacteria.* PCoA plot using Bray-Curtis distance metric shows samples clustering by coccidiosis history (PERMANOVA, *r^2^* = 0.048, *p* = 0.095)(linear model, *r^2^* = 0.1183, *p* = 0.041). Ellipses denote 95% confidence level.

**Supplementary Tables**

**Supplementary Table 1:** Sample collection sites along with the latitude and longitude, captivity status, and sampling size.

**Supplementary Table 2:** *Captivity influences bacterial and fungal communities*. PERMANOVA results for different factors as predictors of microbial variance. Number of asterisks indicate level of statistical significance (****p* < 0.001, ***p* < 0.01, **p* < 0.05).

**Supplementary Table 3:** Clamtest categorizing bacterial and fungal OTUs found in wild and captive kiwi into rare, generalist, wild specialist, and captive specialist.

**Supplementary Table 4:** Most influential bacterial OTUs distinguishing between wild and captive kiwi samples listed by highest contributing OTU in descending order. 13 bacterial OTUs significantly account for over 70% of the differences between captivity status. OTUs that contributed to less than 1% significance was removed. A p-value was calculated per OTU, in addition to false discovery rate (FDR) adjusted p-value. Mean abundance and standard deviation of each OTU is listed between groups.

**Supplementary Table 5:** Most influential fungal OTUs distinguishing between wild and captive kiwi samples listed by highest contributing OTU in descending order. Two fungal OTUs significantly account for over 70% of the differences between captivity status. OTUs that contributed to less than 1% significance were removed. A p-value was calculated per OTU, in addition to false discovery rate (FDR) adjusted p-value. Mean abundance and standard deviation of each OTU is listed between groups.

**Supplementary Table 6:** Clamtest categorizing bacterial and fungal OTUs found in captive kiwi with and without a history of coccidiosis into rare, generalist, positive specialist, and negative specialist.
